# The Importance of Ambient Sound Level to Characterise Anuran Habitat

**DOI:** 10.1371/journal.pone.0078020

**Published:** 2013-10-21

**Authors:** Sandra Goutte, Alain Dubois, Frédéric Legendre

**Affiliations:** Département Systématique et Évolution, MNHN, Paris, France; Università degli Studi di Napoli Federico II, Italy

## Abstract

Habitat characterisation is a pivotal step of any animal ecology study. The choice of variables used to describe habitats is crucial and need to be relevant to the ecology and behaviour of the species, in order to reflect biologically meaningful distribution patterns. In many species, acoustic communication is critical to individuals’ interactions, and it is expected that ambient acoustic conditions impact their local distribution. Yet, classic animal ecology rarely integrates an acoustic dimension in habitat descriptions. Here we show that ambient sound pressure level (SPL) is a strong predictor of calling site selection in acoustically active frog species. In comparison to six other habitat-related variables (i.e. air and water temperature, depth, width and slope of the stream, substrate), SPL had the most important explanatory power in microhabitat selection for the 34 sampled species. Ambient noise was particularly useful in differentiating two stream-associated guilds: torrents and calmer streams dwelling species. Guild definitions were strongly supported by SPL, whereas slope, which is commonly used in stream-associated habitat, had a weak explanatory power. Moreover, slope measures are non-standardized across studies and are difficult to assess at small scale. We argue that including an acoustic descriptor will improve habitat-species analyses for many acoustically active taxa. SPL integrates habitat topology and temporal information (such as weather and hour of the day, for example) and is a simple and precise measure. We suggest that habitat description in animal ecology should include an acoustic measure such as noise level because it may explain previously misunderstood distribution patterns.

## Introduction

Characterising habitats to describe their connection with inhabiting species is fundamental to animal ecology. Ecologists commonly link or predict species occurrences from a combination of biotic and abiotic factors and across micro to macro scales. In any study, the choices of scale and variables describing habitats are crucial and need to be relevant to the ecology and behaviour of the organisms studied [1]. In this characterization, although many variables have become standard, ecologists still rarely consider the acoustic dimension of the habitats.

Acoustic communication is utilised by many species to perform various functions within their populations: to attract potential mates [2,3]; to defend territories [4]; to alert conspecifics to predator presence [5–8]; to maintain social cohesion [9]; to locate prey [10,11]; or to help with orientation [12–14]. The fitness of individuals is directly related to the efficiency of this communication, and external factors influencing acoustic signal transmission play an important role in the ecology of these species. 

Among these factors, the acoustic properties of the physical environment impact sound wave propagation [15–19], with the exact same sound emitted in two different habitats potentially sounding different to identical receivers. For example, acoustic waves are scattered by vegetation and sounds propagate further in open areas than in forests [15,18,20]. The effect of the habitat structure on the acoustic wave also varies according to both sound frequency and the position of the sound source [16,21]. In other words, the environment distorts sounds differently according to its own structure and to the sound structure [16]. In addition, sounds emanating from biotic, abiotic and human sources (constituting the soundscape; [19]) may act as masking noise when considering a particular signal, and impede its detection from the intended receiver(s). 

It is legitimate to include acoustic information in habitat description when studying the ecology of acoustically active species, because ambient acoustic conditions may affect individuals’ interactions, and ultimately individuals’ spatial distribution. In this context, the impact of anthropogenic noise on wildlife has drawn an increasing interest among bioacousticians in the past years, notably with studies on city noise pollution effects on birds [22–27], road noise effects on frogs [28,29] and marine noise effects on fishes and marine mammals [30–32]. Despite these studies, the effect of natural noise on the ecology and evolution of animal species is rarely investigated [33,34], and ambient noise level remains an underused variable in ecological studies [35–39]. We demonstrate here that ambient sound pressure level (SPL), a measure of sound intensity, is a useful descriptor of habitats and illustrate this point with data on Asian frogs. We focus on characterizing frog guilds, and, in particular, on separating ‘torrent’ and calmer stream dwelling species.

‘Torrent frogs’ live and breed in particular habitats where they face a severe acoustic constraint, namely a loud background noise produced by the fast flowing water [40–42]. Recently, the discovery of torrent frogs producing and responding to ultrasounds [43–45] strengthened interest in these frogs and their habitats. However, the study of torrent frogs as a group suffers from two entangled problems: some frog species are not unambiguously considered as ‘torrent frogs’ by all herpetologists and torrents are not unambiguously defined among ecologists. We detail these limits hereafter.

‘Torrent frogs’ is a common phrasing used in the literature defining an ecological unit - or guild [46] - comprising a group of anuran genera without, necessarily, direct phylogenetic relationships [47–49]. It designates animals that show morphological or behavioural traits thought to be adaptive to life (permanently or temporary) in fast flowing water. However, this ecological designation does not always relate to the actual or known ecology of the species, as new species referred to ‘torrent frog’ genera are credited with a torrent dwelling ecology although their life history is largely undocumented. The reverse can also be true, when an actual torrent dwelling frog species does not belong to one of the genera considered as torrent genera. In fact, in some cases, this questions the relevance and validity of the genera as currently recognised by taxonomists.

The torrent habitat is usually characterized by variables such as slope, width and depth of a stream. This set of variables is, however, incomplete because both a steep, swift and narrow stream and a large tumultuous river on a flatter terrain can be considered as ‘torrents’. Moreover, measuring the slope of a stream, which constitutes a key component of torrents description, is problematic with methods varying among studies and scales [36,38,42,50]. 

In this context, an accurate torrent guild definition is impossible without further investigation of the actual ecology of each species. This difficulty hinders interpretations in ecological, behavioural or evolutionary studies. We show here how integrating an acoustic variable to characterize habitats can help in this respect by providing a standardised and more biologically meaningful surrogate to common physical descriptors, especially slope. 

## Materials and Methods

### Data collection

Data were collected at 13 localities within six regions of Southeast Asia: Preah Vihear province, Cambodia, from 11 to 20 December 2010 [authorisation given by the Autorité Nationale pour la Protection et le Dévelopement du Temple de Preah Vihear (ANPV)]; West Kalimantan province, Indonesia, from 9 June to 3 August 2011 (permit delivered by the Direktorat Jenderal Perlindungan Hutan Dan Konservasi Asi Alam, LIPI: 3394/IPH.1/KS.02/VI/2011); Sichuan, Hunan and Hainan provinces, China, from 7 June to 19 July 2012 (we worked in collaboration with Pr. Jiang Jianping at the Chinese Academy of Sciences, who got permits from local province or districtal forest departments on presenting an introduction letter of his institute for data collection); and Sabah province, Malaysia, from 8 to 24 August 2012 (permit delivered by Sabah Parks: 00TS/PTD/5/4 Jld. 46 (43) ). Locality names, GPS coordinates and altitudinal ranges can be found in Table 1. Prior to sampling, local authorities approved all collected species. No endangered species were sampled. 

**Table 1:** Details about the sampled localities.

**Locality**	**Province**	**Country**	**GPS coordinates**	**Min. altitude (m)**	**Max. altitude (m)**
O Kampol Neak	Preah Vihear	Cambodia	N13°49, E104°49	44	44
O Sam Bour	Preah Vihear	Cambodia	N14°22, E104°47	105	105
Emei Shan	Sichuan	China	N29°35' - N29°32', E103°17' - E103°19'	1324	2256
Qing Cheng	Sichuan	China	N30°55' - N30°56', E103°29' - E103°28'	933	1286
Long Quan	Sichuan	China	N30°31' - N30°31', E104°21' - E104°23'	452	468
Jian Yang	Sichuan	China	N30°18', E104°17'	455	455
Zihuai	Sichuan	China	N28°42' - N28°37', E106°15' - E106°18'	764	776
Badagong Shan	Hunan	China	N29°41' - N29°47', E110°04' - E110°07'	380	1399
Puqian Bay	Hainan	China	N19°57', E110°34'	5	5
Rice field	Hainan	China	N19°29', E110°24'	88	88
Diaoluo Shan	Hainan	China	N18°43' - N18°43', E109°52' - E10° 51'	915	944
Bukit Baka - Bukit Raya Nat. Park	West Kalimantan	Indonesia	S0° 43’, E112°16’	60	960
Mount Kinabalu Nat. Park	Sabah	Malaysia	N6°00’, E116°32’	1400	1500

Frogs were sampled from a range of still (such as ponds, lakes, puddles) or running (such as cascades, streams, rivers) freshwater-associated habitats. Calling males were located acoustically and visually at night from 19:00 h to 24:00 h. The call type most often heard from single males was considered to be the advertisement call. Other call types produced by conspecific males, often heard when they were close to each other or during fights, were considered as aggressive or release calls and these calls were disregarded. After recording, specimens were caught by hand, identified to the species level and brought back to the laboratory where they were euthanized using a chlorobutanol solution, measured and fixed for museum collection purposes. The specimens are kept at local museums, respectively: Museum Zoologicum Bogoriense (MZB), Indonesia; Chengdu Institute of Biology (CIB), China; and Sabah Museum (SPM), Malaysia. The specimens from Cambodia are temporarily kept at the Muséum national d’Histoire naturelle, Paris (MNHN), France, but will eventually be hosted in the developing Preah Vihear Museum, Cambodia. 

Once an individual had been captured, habitat descriptors were measured at the exact individual calling post: depth, width and average slope of the closest water body, frog substrate, air and water temperatures, and ambient dBA SPL. For ponds, maximal depth and mean diameter were measured. For streams, maximal depth and width were measured at the focal male level. The slope was averaged over a 10 meter-portion of the stream following the method described in La Perrier and Martin (1986). Briefly, a bendable plastic tube was placed under the water surface, and parallel to the stream course. On its downstream side, the tube was held perpendicularly to the water surface. After stabilisation of the water level in the tube, the height of the water column in the tube was measured. The slope was obtained through the formula: Σ[a_i_]/ Σ [√(c_i_^2-^a_i_^2^)] where a_i_ is the height of the water column in the tube for the i^th^ stream segment, and c_i_ is the length of the immerged part of the tube for the i^th^ segment. The substrate of the focal male while calling was categorised as rock, sand, branch, leaf, leaf-litter, soil and water. Ambient air and water temperature were measured with a probe-K digital thermometer (Hanna). The ambient SPL, in decibels, was measured with a digital SPL meter (American Recorder Technologies), with A-weighting and slow capture (one second averaging). The maximal value for a 30 seconds time window was taken. Flowing water and insects constituted most of the measured background noise. No recordings were conducted under heavy rain or when other frogs were calling in the immediate vicinity. 

Our data set comprised 134 individuals that we identified at the species level (34 species in total) and for which we recorded fine-scale habitat descriptors. Number of individual sampled per species varied form one to twelve. The measurements can be found in the supporting information table S1. In this paper, we generally follow Frost’s [51] taxonomy, with the exception that we recognise *Feihyla vittata* [52] and *Phrynoglossus martensii* [53].

### Statistical Analysis

To assess how habitat descriptors account for species distribution and whether any ‘frog guild’ and notably a ‘torrent guild’ was identifiable, a Multiple Factorial Analysis (MFA) was performed with the FactoMineR package [54,55]within R statistical environment [56]. MFA is similar to the more commonly used Principal Component Analysis (PCA), but allows qualitative and quantitative variables to be included in a single analysis. Here, six quantitative variables (depth, width, slope, SPL, air and water temperatures) and one qualitative variable (frog substrate) were used. These explanatory variables were non-weighted. SPL was expressed in decibel (dB), which is a logarithmic unit based on the ratio of the sound pressure measured in Pascal (Pa) to a reference sound pressure (20 μPa here), because it approached a normal distribution (results of the Shapiro-Wilkinson normality test: W=0.971, p-value=0.00721) more so than in Pascal (W = 0.759, p-value = 2.18e-13). Because normally distributed data are preferable (but not required – [57]) for MFA, the values in the dB scale were used for statistical analyses. Each quantitative variable was zero-centred and scaled to unit standard deviation, to avoid bias from differences in variance of measurements.

An agglomerative hierarchical cluster analysis was subsequently performed on the data from the MFA using the same R package. Whereas MFA is a projection of the data in a multidimensional space, the cluster analysis allows to compare data points and to group them according to their proximity in this space. These two approaches are thus complementary. Besides, performing the cluster analysis on the three first principal components from the MFA instead of raw data allows focusing more thoroughly on the main signal of the data, ultimately resulting in more robust clusters [58]. The clusters were built with Ward’s linkage method [59], to minimise variance within clusters. The optimal partition (i.e. number of clusters) was chosen by minimizing the ratio of two successive partition inter-clusters inertia gains. Clusters can be described through the v-test value of environmental variables and the associated p-value. For categorical variables, the sign of the v-test value indicates whether the category is over- (when the value is positive) or underrepresented (when the value is negative) in the cluster in comparison to the global data. For continuous variables, it indicates whether the mean value for the cluster is over or under the mean value for the whole data. The p-value associated indicates the significance of this over- or underrepresentation. This analysis allows to group individuals into potential ecological guilds and to evaluate the species composition of each guild. These guilds are then used as a proxy to evaluate the importance of different habitat variables in species distribution. The v-test allows the estimation of indicative values of the descriptors for each guild, and thus the assessment of the contribution of each descriptor in the guilds’ definition.

In addition, the same analyses (i.e. MFA and cluster analyses) were run twice more, removing either the SPL or slope variables. This allowed to compare final guild definition and composition and to assess how crucial these two variables are to define microhabitat guilds.

## Results

### MFA

**Figure 1:**
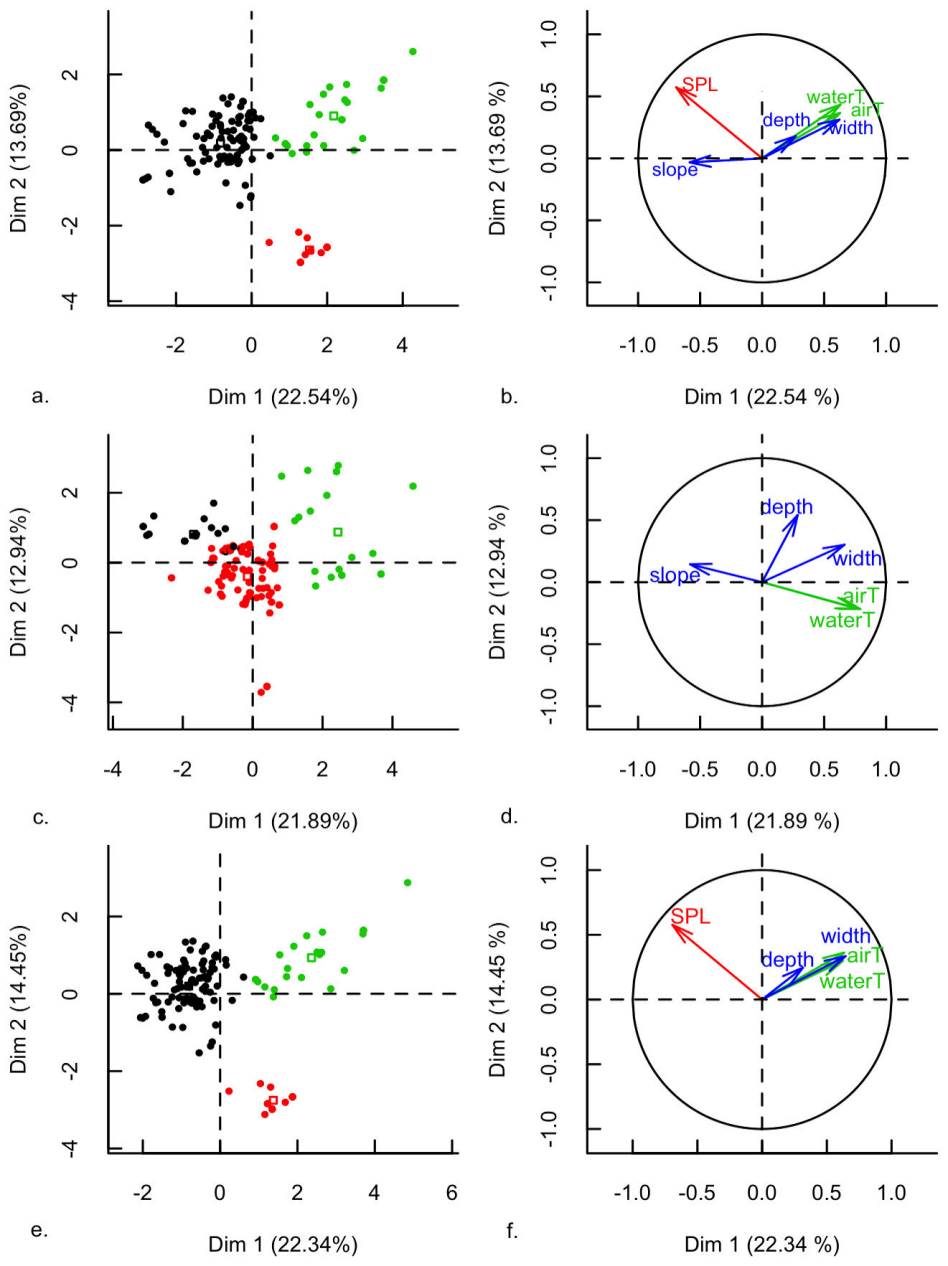
Multiple factorial analysis (MFA) of advertising male frogs microhabitat. a, b. MFA including all variables. c, d. MFA without the SPL variable. e, f. MFA without the slope variable. a, c, e. Mapping of individuals in the first two dimensions resulting from the MFA. Colours of the circles correspond to cluster attribution of the individuals obtained by the hierarchical cluster analysis. Full circles represent the individuals in the MFA space, and open squares the centres of clusters. Black cluster = ‘torrents’, red cluster = ‘ponds’, green cluster = ‘rivers’. b, d, f. Contribution of the quantitative variables to the MFA axes. The length of the vectors corresponds to the amplitude of the variable contribution. Orientations of vectors represent the correlation to the two represented axes. Groups of variables are colour-coded.

The first three dimensions contributed to 47.5 % of the variance in the data. Additional dimensions did not significantly increase the interpretability of the data (cumulative variance with the fourth dimension = 58 %). Therefore, only the first three dimensions were considered. The projection of the data in the first MFA plan (the two first dimensions) is represented on Figure 1a. Ambient noise level (SPL) and water temperature were the best-represented quantitative variables in the MFA (Table 2; Figure 1b). Water and air temperature showed a strong correlation with width and depth of the stream, and were all positively correlated to the first axis, whereas SPL and slope variables were negatively correlated to this axis (Figure 1b). Substrate also had an impact on the species distribution in the MFA space (Table 2): The “water” and “rocks” categories were strongly correlated with the first axis, while the “leaf litter” category was correlated with the second axis. MFA without the SPL variable is displayed in Figure 1 (Fig. 1c = first MFA plan; 1d = variable contributions). It showed a very different structuration than in the MFA with all variables. On the opposite, we found almost no difference when the MFA was run without the slope variable (Figure 1e and 1f: first MFA plan and variable contributions, respectively).

**Table 2:** Contribution (cos^**2**^) of quantitative variables and substrate categories to each MFA dimension.

	**Dim.1**	**Dim.2**	**Dim.3**	**TOTAL**
SPL	0.4862	0.3219	0.0040	0.8121
Water temperature	0.3914	0.1872	0.1681	0.7467
Air temperature	0.3571	0.1267	0.1033	0.5871
Width	0.3857	0.0992	0.0799	0.5648
Slope	0.3378	0.0009	0.0314	0.3701
Depth	0.0736	0.0344	0.1740	0.2820
***Frog substrate***				
Water	0.5156	0.1828	0.1314	0.8298
Leaf litter	0.1735	0.5006	0.0034	0.6775
Ground	0.2496	0.0056	0.2535	0.5087
Rock	0.4782	0.0001	0.0142	0.4925
Sand	0.0669	0.1973	0.226	0.4902
Branch	0.0470	0.0627	0.2173	0.3270
Leaf	0.1094	0.0006	0.0709	0.1809

### Cluster Analysis

**Figure 2:**
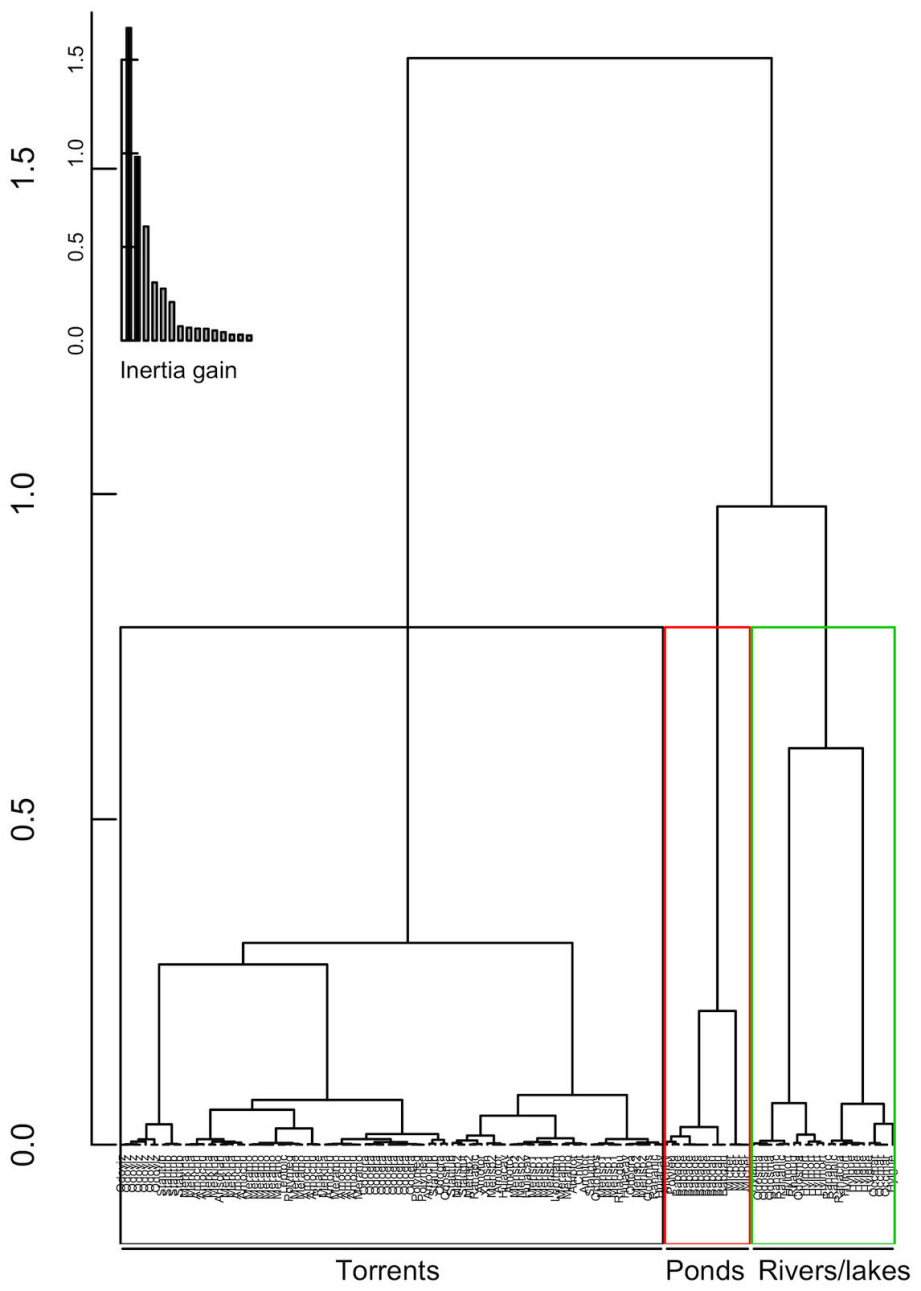
Dendrogram of the individual male frogs resulting from the hierarchical cluster analysis based on their microhabitats. The coloured rectangles correspond to the cluster attribution: black cluster = ‘torrents’, red cluster = ‘ponds’, green cluster = ‘rivers’. Inertia gain for additional dimensions is inset in the top left corner.

We obtained three well-separated clusters (Figure 2). The v-test values and p-values of their environmental variables are summarized in Table 3A and 3B. Given these values, three clusters can be described:

• “Torrents” cluster: very loud, cold, narrow, steep, shallow and rocky streams. This cluster is characterized by “true” torrent dwelling species (i.e. species which are known to have a torrent dwelling ecology): *Meristogenys amoropalamus*, *Odorrana graminea* and *Amolops chunganensis*.
• “Ponds” cluster: quiet, flat, shallow, small water bodies with important leaf litter accumulations, sandy and poorly vegetated sides. This cluster is characterized by pond dwelling species: *Babina adenopleura*, *Microhyla berdmorei* and *Babina daunchina*.
• “Rivers/lakes” cluster: very large, warm, and quieter than torrents but louder than ponds, deep and flat. This corresponds to large rivers, lake networks or rice fields, characterized by the species *Hylarana mortenseni, Hylarana guentheri, Odorrana schmackeri* and *Phrynoglossus martensii.*

**Table 3:** Descriptive values of habitat variables for the three clusters.

A. Continuous variables														
	**TORRENTS**					**PONDS**					**RIVERS / LAKES**			
	**Mean**	**SD**	**v.test**	**p.value**		**Mean**	**SD**	**v.test**	**p.value**		**Mean**	**SD**	**v.test**	**p.value**
SPL	64.51	7.45	8.11	<0.001		45.39	4.37	-6.26	<0.001		52.31	4.05	-4.47	<0.001
Air temperature	19.93	3.17	-4.87	<0.001		22.09	2.72	1.51	0.130		23.59	2.77	4.50	<0.001
Water temperature	19.46	2.92	-5.25	<0.001		21.28	2.63	0.96	0.334		23.80	3.44	5.39	<0.001
Depth	43.40	25.07	-2.58	0.010		40.13	33.18	-0.94	0.343		75.08	64.27	3.79	<0.001
Width	484.74	284.32	-5.00	<0.001		484.13	221.83	-1.14	0.245		1198.08	679.50	6.82	<0.001
Slope	0.20	0.36	3.27	0.001		0.00	0.00	-1.82	0.068		0.01	0.02	-2.36	0.018
B. Substrate categories														
	**TORRENTS**					**PONDS**					**RIVERS / LAKES**			
	**Cat/Clu**	**Clu/Cat**	**v.test**	**p.value**		**Cat/Clu**	**Clu/Cat**	**v.test**	**p.value**		**Cat/Clu**	**Clu/Cat**	**v.test**	**p.value**
Leaf	100	36.17	4.85	<0.001		0	0	-2.35	0.019		0	0	-3.46	<0.001
Rock	100	23.40	3.57	<0.001		0	0	-1.58	0.115		0	0	-2.47	0.014
Branch	90.48	40.43	3.46	<0.001		0	0	-2.82	0.005		9.52	16	-1.63	0.102
Sand	0	0	-2.45	0.014		100	26.67	3.70	<0.001		0	0	-0.17	0.866
Ground	0	0	-4.28	<0.001		0	0	-0.44	0.663		100	36	5.27	<0.001
Leaf litter	0	0	-4.87	<0.001		100	73.33	7.19	<0.001		0	0	-1.32	0.187
Water	0	0	-5.15	<0.001		0	0	-0.75	0.450		100	48	6.33	<0.001

A. Mean and standard deviation values (SD) of each variable are shown for the ’torrents’, ’ponds’ and ‘rivers/lakes’ clusters. B. Percentage of categories’ individuals in each cluster (Cat/Clu) and percentage of clusters’ individuals in each category (Clu/Cat) are represented. The sign of the v-test values indicate whether the variable is over (positive value) or under-represented (negative value) compared to the global average values. P-values of the respective v-tests are represented.

While the water temperature best characterized the ‘rivers/lakes’ cluster, the SPL best characterized the ‘torrents’ and the ‘ponds’ clusters. The SPL range of the ‘torrents’ cluster did not overlap with the SPL range of the two other clusters (Table 3A): a threshold around 56 dB at the frog location separated the torrents cluster. Males were found calling on different substrates in the different microhabitats: rock and leaf for the “torrents” guild; sand and leaf litter for the “pond” guild; ground and in the water for the “rivers and lakes” guild.

Compared to the full analysis, the cluster analysis on MFA without the SPL variable resulted in a less clear separation between clusters, whereas the analysis without the slope variable resulted in comparable results (Figure 1c and Figure 1e).

In the full analysis, cluster attribution of each species fitted the general natural history knowledge of the sampled species (Table 4) with a few notable exceptions that we discuss in the next section 3 species (*Odorrana schmackeri, Hylarana picturata* and *Hylarana nicobariensis*) did not have a clear cluster affiliation, some individuals being present in two different clusters in all three analyses. Removing the SPL variable brought the number of species without a clear cluster affectation to nine, and changed the cluster affiliation for 16 species, whereas removing the slope variable only changed the affiliation from the full analysis of a single individual. Species with highly specific breeding microhabitat preferences, for example *Odorrana yizhangensis* which was only found near a single waterfall, were attributed to two different clusters when not considering the SPL.

**Table 4:** Cluster attribution to the different species.

	**Complete**				**Without SPL**				**Without slope**		
**Species**	**"Torrents"**	**"Ponds"**	**"Rivers / lakes"**		**Clu. 1**	**Clu. 2**	**Clu. 3**		**Clu. A**	**Clu. B**	**Clu. C**
*Amolops chunganensis*	11				8	3*			11		
*Amolops torrentis*	5				5				5		
*Ansonia hanitschi*	1				1				1		
*Babina adenopleura*		6			6*					6	
*Babina daunchina*		3			3*					3	
*Feihyla vittata*	1				1				1		
*Huia cavitympanum*	4				4				4		
*Hyla annectans*			1		1*				1*		
*Hylarana chalconota*			1				1				1
*Hylarana guentheri*			5				5				5
*Hylarana mortenseni*			6		1*		5*				6
*Hylarana nicobariensis*†	1		3		3*		1*		1		3
*Hylarana picturata*†	1		1			1*	1		1		1
*Leptolalax hamidi*	1				1				1		
*Leptolalax pictus*	1				1				1		
*Meristogenys amoropalamus*	12				12				12		
*Meristogenys kinabaluensis*	7				2*	5*			7		
*Meristogenys sp. a*	7				7				7		
*Meristogenys sp. b*	7				5*	2*			7		
*Microhyla berdmorei*		4			4*					4	
*Microhyla heymonsi*		1			1*					1	
*Odorrana graminea*	11				11				11		
*Odorrana hosii*	2				2				2		
*Odorrana schmackeri*†	1		4		4*		1*		1		4
*Odorrana yizhangensis*	7				2*	5*			7		
*Phrynoglossus martensii*			3				3				3
*Polypedates leucomystax*		1			1*					1	
*Polypedates megacephalus*	3				3				3		
*Rhacophorus chenfui*	1				1				1		
*Rhacophorus dugritei*			1				1				1
*Rhacophorus gauni*	1				1				1		
*Staurois guttatus*	2				1*	1*			2		
*Staurois tuberilinguis*	6				1*	5*			6		
*Xenophrys sangzhiensis*	1				1				1		
**TOTAL**	**94**	**15**	**25**		**94**	**22**	**18**		**95**	**15**	**24**

Left panel: complete analysis. Centre and right panels: analyses without the SPL and slope variables, respectively. The numbers of individuals in each cluster are indicated in each cell. The symbol † after the species name indicates that individuals of that species were attributed two different clusters in the complete analysis, and the symbol * indicates a difference of number in individuals in the cluster compared to the complete analysis.

## Discussion

The choice of variables biologically relevant to the studied organisms for describing habitats is crucial in any study design and will determine whether the biological question(s) being asked can be answered. In order to optimize time in the field and information content of measurements, a careful examination of each included variable is needed. We discuss here the value of an acoustic variable, the ambient noise level, in habitat description and guild characterization for ecological studies.

In this study, we considered seven environmental variables and defined three guilds of frog species associated to: torrents, large rivers and lakes, and ponds. These three guilds were clearly identified in MFA, wherein ambient noise level (SPL) was the first discriminating variable, indicating its large contribution to guild characterisation. 

We found the slope variable correlated with SPL (Figure 1b), which was consistent with flowing water being the major noise source in our dataset By running the MFA without the slope variable and obtaining virtually unchanged results (Figure 1e), we showed that, in this dataset, the information brought by the slope was partly redundant with other variables (and in particular SPL) and not necessary for guild definition. Slope is a difficult and non-standardized measure among ecologists [36,38,42,50], especially at a fine scale. As ambient noise ‘carried’ some information about stream slope and is a much simpler measure to standardize, we argue that, in freshwater habitats, the slope variable could be discarded from the environmental variables set if SPL was included. 

Most interestingly, removing the SPL variable from the analysis resulted in different and less clearly discriminated clusters, emphasizing the information brought by ambient noise level. In this configuration, more species were attributed to multiple guilds. While a slope of a given stream is a fixed value, SPL is a ‘dynamic’ one, depending on the habitat topography and on many temporally fluctuating factors (for example: the hour of the day, the season, and the amount of precipitations). Including ambient noise level thus integrates precise temporal information of prime importance for biological organisms. Moreover, whereas slope has to be averaged over a certain distance (10 metres in this study), SPL is measured at the exact calling post of the individual and can reflect differences in microhabitats centimetres away from each other.

As anurans rely heavily on acoustics for reproduction, ambient noise level is of direct relevance regarding their behavioural ecology and ultimately impacts on species distribution. In streams in particular, anurans face a noise constraint from the flowing water. This noise is broad-banded, with a highest energy generally below 1.5kHz (S.Goutte, unpublished data), and could be as loud as 87.9 dB in this dataset. When two sounds have overlapping frequencies, they interfere with one-another, and can partially or completely mask one-another[42,60–62]. As most frog species call at frequencies below 5 kHz [63–66], the noise produced by flowing water can mask their vocalizations.

Some torrent species overcome masking by having evolved a particular call structure [40–42,67,68], higher pitched notes [44,45,69], visual signalling complementing the acoustical signalling [68,70] or ear tuning which acts as a frequency band filter and removes part of the noise [71]. These presumed adaptations open new, unoccupied “acoustic niches” to these species, and our data are congruent with the hypothesis that males choose their calling posts according to, for a part, the acoustic component of the microhabitat. For example, in this study and within streams, a 56 dB threshold for noise level separated the calm (‘lake/rivers’ cluster) and noisy (‘torrent’ cluster) “acoustic niches”, and very few species were found in both habitat types. However, beyond the “acoustic niche” hypothesis (see for example [72]), ambient noise could also carry information about habitat conditions, such as the suitability for egg laying, that have not been evaluated here. Given the importance of acoustic communication in anurans and in many other groups, it is difficult to understand why SPL is not yet a common variable in habitat description for animal ecology studies. While more in-depth analyses of the acoustic environment is time consuming both in a field and the lab, SPL is a rapid and precise measurement readily useable for ecologists.

Although including SPL into our habitat variables allowed a clear separation of three guilds, we did find “surprising” results in guild composition (see Table 4): some individuals were assigned to unexpected clusters in regard to their phylogenetic group or to the general knowledge of their ecology, and a few species had individuals in two different guilds. Like every scientific result, these unexpected outcomes are all the more intriguing and worth discussing that the samples are large. Nevertheless, even species with a low sample can draw attention on potentially valuable pitfalls or discoveries (see for instance *Feihyla vittata* below). Thus, although *Odorrana schmackeri* (with common name for *Odorrana* being “torrent frogs”) is a species “automatically” attributed a torrent dwelling life due to its place in the phylogenetic tree, we found males solely in large but generally quiet and rather flat streams. As a result, most individuals of this species were assigned the ‘rivers/lakes’ guild. As for *Hylarana picturata*, its known natural history places calling males in calm streams, corresponding also to the ‘rivers/lakes’ guild. However, for both *H. picturata* and *O. schmackeri*, one individual was classified in the ‘torrents’ guild. This might illustrate a variability of choice in calling post for those species, for which other unmeasured variables might be of importance, reflect marginal behaviours, or more simply, suggest that our categorization does not reflect exactly these two species microhabitat selection. Unfortunately, the sample size for these species does not allow further comments on this aspect. In addition, three individuals of the common pond species *Polypedates megacephalus* and one individual of *Feihyla vittata* were attributed the ‘torrents’ guild. These individuals were recorded in small pools, separated but very close to a large fast flowing stream. The environmental measurements taken for these individuals have thus inevitably involved some of the large stream characteristics (such as the ambient noise). Finally, the attribution to the ‘torrents’ cluster of one individual of *Hylarana nicobariensis* is explained by a combined elevated SPL and a less common substrate (branch) for that habitat type. In this general context, SPL must be considered in combination with other habitat descriptors.

Classifying complex biological phenomenon into a limited number of discrete categories is often necessary to understand underlying biological processes, and although our analysis presents a categorisation of the 34 sampled frog species into three guilds, this classification, like any classification, still represents a simplification of a complex reality [73]. Our definition of the three freshwater habitats types is based on seven habitat descriptors, and interactions between these descriptors and other habitat variables are likely. The classification we propose here should thus be regarded as a conceptual framework for further testing ecological and evolutionary hypotheses, accounting for the importance of the acoustic dimension to species-habitat interactions.

## Conclusions

Ambient sound level affects a whole range of acoustically active species, affecting in turn dependent species. Although the number of studies on anthropogenic noise pollution effects on fauna is increasing, ambient sound level is too often forgotten in natural habitat community ecology studies. The success of classical microhabitat variables in explaining species distribution is often mitigated and authors observe ‘suitable habitats’ oddly emptied of the expected species [39]. While explanatory power of analyses may be lowered due to random variations in the samples, we believe that adding an ambient noise measure may improve microhabitats occupancy predictions. SPL is a precise, quick, inexpensive and easy measure that could be used to produce more biologically informative habitat typologies and guild characterization in many studies directed towards acoustically active species.

## Supporting Information

Table S1 **Values for habitat variables for each individual.**
(DOC)
