# Supplementary material for: The Importance of Ambient Sound Level to Characterise Anuran Habitat
Source: PLoS One. 2013 Oct 21;8(10):e78020. doi: 10.1371/journal.pone.0078020 (PMC3804622; doi:10.1371/journal.pone.0078020)
Supplement: Table S1 — Values for habitat variables for each individual. (DOC) [file pone.0078020.s001.doc]

| **Country** | **Site** | **Specimen field number** | **Genus** | **Species** | **SPL (dB)** | **Substrate** | **Air temperature (°C)** | **Water temperature (°C)** | **Depth (cm)** | **Width (cm)** | **Slope (%)** |
| --- | --- | --- | --- | --- | --- | --- | --- | --- | --- | --- | --- |
| China | Badagong Shan | 2301 | Amolops | chunganensis | 44.1 | leaf | 17.5 | 15.2 | 9 | 233 | 0,0180 |
| China | Badagong Shan | 2310 | Amolops | chunganensis | 52.5 | branch | 17.2 | 15.2 | 16 | 111 | 0,0120 |
| China | Badagong Shan | 2403 | Amolops | chunganensis | 43.9 | leaf | 18.8 | 17.5 | 7 | 299 | 0,0180 |
| China | Qing Cheng | NA | Amolops | chunganensis | 61.9 | rock | 17.6 | 16.1 | 96 | 470 | 0,0250 |
| China | Qing Cheng | 70103 | Amolops | chunganensis | 64 | rock | 17.6 | 16.1 | 48 | 378 | 0,0217 |
| China | Qing Cheng | NA | Amolops | chunganensis | 60.2 | rock | 17.6 | 16.1 | 48 | 264 | 0,0217 |
| China | Qing Cheng | 70104 | Amolops | chunganensis | 60.1 | branch | 17.6 | 16.1 | 55 | 423 | 0,0350 |
| China | Qing Cheng | 70105 | Amolops | chunganensis | 58.1 | branch | 17.6 | 16.1 | 55 | 423 | 0,0350 |
| China | Qing Cheng | 70106 | Amolops | chunganensis | 59.4 | branch | 17.6 | 16.1 | 42 | 701 | 0,0947 |
| China | Qing Cheng | 70107 | Amolops | chunganensis | 63 | branch | 17.6 | 16.1 | 35 | 71.4 | 0,0947 |
| China | Qing Cheng | 70108 | Amolops | chunganensis | 61.8 | branch | 17.6 | 16.1 | 53 | 280 | 0,0217 |
| China | Diaoluo Shan | 0026H | Amolops | torrentis | 71.8 | leaf | 22.8 | 21.6 | 12 | 303 | 0,0482 |
| China | Diaoluo Shan | 0033H | Amolops | torrentis | 63 | leaf | 22.8 | 21.6 | 21 | 456 | 0,0619 |
| China | Diaoluo Shan | 0023H | Amolops | torrentis | 61.1 | leaf | 24.9 | 21.6 | 21 | 646 | 0,0467 |
| China | Diaoluo Shan | NA | Amolops | torrentis | 67.9 | leaf | 24.1 | 21.4 | 26 | 251 | 0,1560 |
| China | Diaoluo Shan | 0040H | Amolops | torrentis | 73.2 | branch | 24.1 | 21.4 | 22 | 262 | 0,4651 |
| Malaysia | Kinabalu NP | 0051H | Ansonia | hanitschi | 65.2 | leaf | 17.4 | 17.7 | 130 | 379 | 0,7561 |
| China | Diaoluo Shan | NA | Babina | adenopleura | 42.3 | leaf litter | 24.9 | 21.6 | 20 | 759 | 0,0000 |
| China | Diaoluo Shan | NA | Babina | adenopleura | 42.3 | leaf litter | 24.9 | 21.6 | 20 | 759 | 0,0000 |
| China | Diaoluo Shan | NA | Babina | adenopleura | 42.3 | leaf litter | 24.9 | 21.6 | 20 | 759 | 0,0000 |
| China | Diaoluo Shan | NA | Babina | adenopleura | 42.3 | leaf litter | 24.9 | 21.6 | 20 | 759 | 0,0000 |
| China | Diaoluo Shan | NA | Babina | adenopleura | 41.5 | leaf litter | 24.9 | 21.6 | 53 | 238 | 0,0000 |
| China | Diaoluo Shan | NA | Babina | adenopleura | 49.2 | leaf litter | 24.9 | 21.6 | 53 | 238 | 0,0000 |
| China | Emei Shan | 611 | Babina | daunchina | 43.8 | leaf litter | 18 | 17 | 100 | 610 | 0,0000 |
| China | Emei Shan | 609 | Babina | daunchina | 43.8 | leaf litter | 18 | 17 | 100 | 610 | 0,0000 |
| China | Emei Shan | 610 | Babina | daunchina | 43.8 | leaf litter | 18 | 17 | 100 | 610 | 0,0000 |
| China | Diaoluo Shan | 0031H | Feihyla | vittata | NA | leaf | 22.8 | 21.6 | 5 | 247 | 0,0000 |
| Indonesia | Bukit Baka - Bukit Raya | 2278 | Huia | cavitympanum | 68.3 | leaf | 22.9 | 23 | 50 | 536 | 0,0894 |
| Indonesia | Bukit Baka - Bukit Raya | 2283 | Huia | cavitympanum | 68.3 | branch | 22.9 | 23 | 50 | 455 | 0,1009 |
| Indonesia | Bukit Baka - Bukit Raya | 2290 | Huia | cavitympanum | 66 | leaf | 23 | 22.8 | 110 | 840 | 0,1040 |
| Indonesia | Bukit Baka - Bukit Raya | 2341 | Huia | cavitympanum | 74.4 | leaf | 22.4 | 22.8 | 32 | 470 | 0,2082 |
| China | Zihuai | 61004 | Hyla | annectans | 56 | branch | 20.6 | 18 | 12 | 2000 | 0,0000 |
| China | Long Quan | NA | Hylarana | guentheri | 55.4 | water | 26.7 | 25.7 | 120 | 600 | 0,0000 |
| China | Long Quan | 605 | Hylarana | guentheri | 49.6 | water | 25.9 | 27.2 | 75 | 300 | 0,0000 |
| China | Long Quan | 606 | Hylarana | guentheri | 50.9 | water | 25.9 | 27.2 | 75 | 300 | 0,0000 |
| China | Long Quan | 607 | Hylarana | guentheri | 50.5 | water | 25.9 | 28.3 | 300 | 2134 | 0,0000 |
| China | Long Quan | NA | Hylarana | guentheri | 47.4 | water | 26 | 25.9 | 75 | 1852 | 0,0000 |
| Cambodia | O Sam Bour | K4912 | Hylarana | mortenseni | 59 | water | 23.3 | 24.1 | 23 | 1360 | 0,0808 |
| Cambodia | O Sam Bour | K4911 | Hylarana | mortenseni | 43.2 | ground | 23.3 | 24.1 | 130 | 1500 | 0,0000 |
| Cambodia | O Sam Bour | NA | Hylarana | mortenseni | 55 | ground | 23.3 | 24.1 | 130 | 1500 | 0,0000 |
| Cambodia | O Sam Bour | NA | Hylarana | mortenseni | 55 | ground | 23.3 | 24.1 | 130 | 1500 | 0,0000 |
| Cambodia | O Sam Bour | NA | Hylarana | mortenseni | 56.5 | ground | 24.7 | 22.9 | 19 | 960 | 0,0582 |
| Cambodia | O Sam Bour | NA | Hylarana | mortenseni | 44.5 | ground | 23.5 | 23.7 | 150 | 1500 | 0,0030 |
| Indonesia | Bukit Baka - Bukit Raya | 2238 | Leptolalax | hamidi | 65.4 | leaf | 24.1 | 23.7 | 38 | 1058 | 0,0140 |
| Malaysia | Kinabalu NP | 0058H | Leptolalax | pictus | 58.8 | leaf | 17.6 | 17.4 | 95 | 378 | 0,0501 |
| Malaysia | Kinabalu NP | NA | Meristogenys | amoropalamus | 59.1 | leaf | NA | NA | 41 | 244 | 0,0667 |
| Malaysia | Kinabalu NP | 0049H | Meristogenys | amoropalamus | 59.7 | leaf | 17.4 | 17.7 | 31 | 852 | 0,0338 |
| Malaysia | Kinabalu NP | 0050H | Meristogenys | amoropalamus | 57.3 | leaf | 17.4 | 17.7 | 40 | 847 | 0,0338 |
| Malaysia | Kinabalu NP | 0053H | Meristogenys | amoropalamus | 62.3 | leaf | 16.5 | 17 | 38 | 797 | 0,0338 |
| Malaysia | Kinabalu NP | 0052H | Meristogenys | amoropalamus | 61.6 | leaf | 16.5 | 17 | 27 | 531 | 0,0338 |
| Malaysia | Kinabalu NP | 0056H | Meristogenys | amoropalamus | 62 | leaf | 17.3 | 17.1 | 48 | 532 | 0,0667 |
| Malaysia | Kinabalu NP | 0066H | Meristogenys | amoropalamus | 60 | leaf | NA | NA | NA | 658 | 0,0338 |
| Malaysia | Kinabalu NP | 0068H | Meristogenys | amoropalamus | 63.1 | branch | 16.2 | 17.3 | 45 | 826 | 0,0338 |
| Malaysia | Kinabalu NP | 0069H | Meristogenys | amoropalamus | 62.7 | leaf | 16.2 | 17.3 | 45 | 826 | 0,0338 |
| Malaysia | Kinabalu NP | 0076H | Meristogenys | amoropalamus | 63.1 | leaf | 15.3 | 17.5 | 45 | 811 | 0,0338 |
| Malaysia | Kinabalu NP | 0077H | Meristogenys | amoropalamus | 59.8 | leaf | 15.3 | 17.5 | 68 | 723 | 0,0338 |
| Malaysia | Kinabalu NP | NA | Meristogenys | amoropalamus | 58.1 | branch | 16.3 | 17 | 27 | 843 | 0,0338 |
| Indonesia | Bukit Baka - Bukit Raya | 2236 | Meristogenys | sp.a | 72.9 | branch | 24.1 | 23.7 | 21 | 821 | 0,0506 |
| Indonesia | Bukit Baka - Bukit Raya | 2237 | Meristogenys | sp.a | 66.9 | leaf | 24.1 | 23.7 | 21 | 821 | 0,0506 |
| Indonesia | Bukit Baka - Bukit Raya | 2239 | Meristogenys | sp.a | 69.1 | leaf | 23.6 | 23.7 | 25 | 937 | 0,0270 |
| Indonesia | Bukit Baka - Bukit Raya | 2242 | Meristogenys | sp.a | 70.4 | leaf | 23.3 | 22.9 | NA | NA | 0,0380 |
| Indonesia | Bukit Baka - Bukit Raya | 2247 | Meristogenys | sp.a | 74.8 | branch | 24 | 23.3 | 30 | 397 | 0,1219 |
| Indonesia | Bukit Baka - Bukit Raya | 2253 | Meristogenys | sp.a | 71.6 | branch | 23.3 | 23.7 | 48 | 541 | 0,0295 |
| Indonesia | Bukit Baka - Bukit Raya | 2347 | Meristogenys | sp.a | 67.3 | leaf | 23 | 23.7 | 49 | 720 | 0,0320 |
| Indonesia | Bukit Baka - Bukit Raya | 2227 | Meristogenys | sp.b | 74 | leaf | 23.2 | 22.8 | 28 | 58 | 0,0888 |
| Indonesia | Bukit Baka - Bukit Raya | NA | Meristogenys | sp.b | 80.1 | rock | 23.2 | 22.8 | 47 | 58 | 0,0888 |
| Indonesia | Bukit Baka - Bukit Raya | 2228 | Meristogenys | sp.b | 67.6 | branch | 22.1 | 22.8 | 53 | 941 | 0,0923 |
| Indonesia | Bukit Baka - Bukit Raya | 2229 | Meristogenys | sp.b | 67.8 | branch | 22.1 | 22.8 | 30 | 1049 | 0,0421 |
| Indonesia | Bukit Baka - Bukit Raya | 2230 | Meristogenys | sp.b | 69.9 | rock | 22.1 | 22.8 | 36 | 610 | 0,0728 |
| Indonesia | Bukit Baka - Bukit Raya | 2241 | Meristogenys | sp.b | 70.7 | leaf | 23.3 | 22.9 | 24 | 464 | 0,0431 |
| Indonesia | Bukit Baka - Bukit Raya | 2342 | Meristogenys | sp.b | 66.8 | leaf | 22.4 | 22.8 | 32 | 470 | 0,1785 |
| Malaysia | Kinabalu NP | NA | Meristogenys | kinabaluensis | 67.5 | rock | NA | NA | 95 | 378 | 0,0501 |
| Malaysia | Kinabalu NP | 0061H | Meristogenys | kinabaluensis | 64.3 | rock | 17.6 | 17.4 | 52 | 869 | 0,0360 |
| Malaysia | Kinabalu NP | 0062H | Meristogenys | kinabaluensis | 70.3 | rock | 17.3 | 17.8 | 150 | 515 | 0,0989 |
| Malaysia | Kinabalu NP | 0063H | Meristogenys | kinabaluensis | 71.4 | branch | 16.9 | 17.9 | 62 | 389 | 0,0501 |
| Malaysia | Kinabalu NP | 0073H | Meristogenys | kinabaluensis | 64.9 | branch | 16.8 | 17.6 | 55 | 361 | 0,0501 |
| Malaysia | Kinabalu NP | 0074H | Meristogenys | kinabaluensis | 66 | rock | 16.8 | 17.6 | 46 | 309 | 0,0501 |
| Malaysia | Kinabalu NP | 0075H | Meristogenys | kinabaluensis | 64.1 | rock | 16.7 | 17.3 | 46 | 309 | 0,0501 |
| Cambodia | O Kampol Neak | K4977 | Microhyla | berdmorei | 45 | sand | 21.4 | 23.7 | 23 | 410 | 0,0000 |
| Cambodia | O Kampol Neak | K4980 | Microhyla | berdmorei | 45 | sand | 21.4 | 23.7 | 23 | 410 | 0,0000 |
| Cambodia | O Kampol Neak | K4981 | Microhyla | berdmorei | 45 | sand | 21.4 | 23.7 | 23 | 410 | 0,0000 |
| Cambodia | O Kampol Neak | K4982 | Microhyla | berdmorei | 45 | sand | 21.4 | 23.7 | 12 | 210 | 0,0000 |
| China | Zihuai | 61002 | Microhyla | heymonsi | 56.8 | leaf litter | 20.1 | 19 | 20 | 180 | 0,0000 |
| China | Rice field | 0035H | Phrynoglossus | martensii | 49.4 | water | 27.8 | 28 | 13 | 2000 | 0,0000 |
| China | Rice field | 0036H | Phrynoglossus | martensii | 49.4 | water | 27.8 | 28 | 13 | 2000 | 0,0000 |
| China | Rice field | 0039H | Phrynoglossus | martensii | 49.4 | water | 27.8 | 28 | 13 | 2000 | 0,0000 |
| China | Zihuai | 61005 | Odorrana | graminea | 53.4 | branch | 20.6 | 18 | 87 | 280 | 0,0025 |
| China | Zihuai | 61006 | Odorrana | graminea | 58.7 | branch | 20.6 | 18 | 87 | 280 | 0,0025 |
| China | Zihuai | 61007 | Odorrana | graminea | 67 | branch | 19.7 | 18.6 | 38 | 291 | 0,0105 |
| China | Zihuai | 61008 | Odorrana | graminea | 61.8 | branch | 19.7 | 18.6 | 55 | 862 | 0,0407 |
| China | Zihuai | 61009 | Odorrana | graminea | 63.1 | branch | 19.7 | 18.6 | 55 | 862 | 0,0407 |
| China | Zihuai | 61101 | Odorrana | graminea | 63.3 | branch | 20.8 | 17.6 | 59 | 466 | 0,0484 |
| China | Zihuai | 61102 | Odorrana | graminea | 64.8 | branch | 20.8 | 17.6 | 59 | 466 | 0,0484 |
| China | Zihuai | 61103 | Odorrana | graminea | 69 | branch | 19 | 17.3 | 56 | 662 | 0,0088 |
| China | Zihuai | 61107 | Odorrana | graminea | 63.4 | branch | 19 | 17.7 | 54 | 934 | 0,0088 |
| China | Zihuai | 61108 | Odorrana | graminea | 62.1 | branch | 19 | 17.7 | 55 | 1006 | 0,0088 |
| China | Diaoluo Shan | 0042H | Odorrana | graminea | 63.4 | branch | 24.1 | 21.4 | 58 | 331 | 0,1260 |
| Indonesia | Bukit Baka - Bukit Raya | 2345 | Odorrana | hosii | 67.4 | branch | 23 | 23.7 | 19 | 975 | 0,0380 |
| Indonesia | Bukit Baka - Bukit Raya | toeclip | Odorrana | hosii | 76.8 | branch | 22.9 | 23.5 | 38 | 680 | 0,0380 |
| China | Badagong Shan | 1901 | Odorrana | schmackeri | 51.3 | branch | 20.3 | 18.7 | 100 | 1547 | 0,0025 |
| China | Badagong Shan | 1904 | Odorrana | schmackeri | 51.3 | branch | 20.3 | 18.7 | 100 | 1547 | 0,0025 |
| China | Badagong Shan | 1903 | Odorrana | schmackeri | 51.9 | ground | 20.3 | 18.7 | 100 | 1547 | 0,0025 |
| China | Badagong Shan | 1902 | Odorrana | schmackeri | 50.2 | branch | 20.3 | 18.7 | 100 | 1547 | 0,0025 |
| China | Badagong Shan | 2001 | Odorrana | schmackeri | 50.3 | branch | 22.4 | 21 | 50 | 485 | 0,0025 |
| China | Badagong Shan | 2302 | Odorrana | yizhangensis | 53 | rock | 15.5 | 15.2 | 60 | 378 | 1,2085 |
| China | Badagong Shan | 2303 | Odorrana | yizhangensis | 62.7 | rock | 15.5 | 15.2 | 41 | 5 | 1,2085 |
| China | Badagong Shan | 2304 | Odorrana | yizhangensis | 62.7 | rock | 15.5 | 15.2 | 41 | 5 | 1,2085 |
| China | Badagong Shan | 2311 | Odorrana | yizhangensis | 62.6 | rock | 17.2 | 15.2 | 15 | 57 | 1,2085 |
| China | Badagong Shan | 2312 | Odorrana | yizhangensis | 62.6 | leaf | 17.2 | 15.2 | 15 | 160 | 1,2085 |
| China | Badagong Shan | 2313 | Odorrana | yizhangensis | 62.6 | rock | 17.2 | 15.2 | 15 | 160 | 1,2085 |
| China | Badagong Shan | 2314 | Odorrana | yizhangensis | 62.6 | leaf | 17.2 | 15.2 | 15 | 160 | 1,2085 |
| Indonesia | Bukit Baka - Bukit Raya | 2270 | Polypedates | leucomystax | 52.7 | leaf litter | 22.3 | 24.8 | 15 | 300 | 0,0000 |
| China | Zihuai | NA | Polypedates | megacephalus | NA | branch | 20.1 | 19 | NA | NA | 0,0000 |
| China | Zihuai | NA | Polypedates | megacephalus | NA | branch | 20.1 | 19 | NA | NA | 0,0000 |
| China | Zihuai | 61003 | Polypedates | megacephalus | 55.9 | branch | 20.1 | 19 | 20 | 180 | 0,0000 |
| Indonesia | Bukit Baka - Bukit Raya | 2306 | Hylarana | chalconota | 57.8 | water | 26 | 24.5 | 60 | 264 | 0,0000 |
| Indonesia | Bukit Baka - Bukit Raya | NA | Hylarana | nicobariensis | 54.5 | ground | 22.5 | 25.3 | 30 | 300 | 0,0000 |
| Indonesia | Bukit Baka - Bukit Raya | 2260 | Hylarana | nicobariensis | 62.5 | branch | 22.5 | 25.3 | 15 | 250 | 0,0000 |
| Indonesia | Bukit Baka - Bukit Raya | 2275 | Hylarana | nicobariensis | 52.7 | ground | 22.3 | 24.8 | 15 | 300 | 0,0000 |
| Indonesia | Bukit Baka - Bukit Raya | 2276 | Hylarana | nicobariensis | 52.7 | water | 22.3 | 24.8 | 15 | 300 | 0,0000 |
| Indonesia | Bukit Baka - Bukit Raya | 2299 | Hylarana | nicobariensis | 68.8 | rock | 24 | 23.2 | 44 | 472 | 0,0000 |
| Indonesia | Bukit Baka - Bukit Raya | 2305 | Hylarana | picturata | 57.8 | water | 21.6 | 23.8 | 60 | 264 | 0,0000 |
| Indonesia | Bukit Baka - Bukit Raya | NA | Rhacophorus | gauni | 72.9 | branch | 23 | 23.7 | 19 | 975 | 0,0380 |
| China | Badagong Shan | 2024 | Rhacophorus | chenfui | 44.8 | leaf | 17.5 | 16.4 | 10 | 642 | 0,0000 |
| China | Emei Shan | 607 | Rhacophorus | dugritei | 56.3 | ground | 18 | 17.8 | 100 | 830 | 0,0000 |
| Indonesia | Bukit Baka - Bukit Raya | 2291 | Staurois | guttatus | 77.8 | branch | 23.7 | 22.5 | 56 | 380 | 0,1030 |
| Indonesia | Bukit Baka - Bukit Raya | 2343 | Staurois | guttatus | 78.6 | rock | 23.3 | 22.5 | 37 | 341 | 0,1009 |
| Indonesia | Bukit Baka - Bukit Raya | NA | Staurois | tuberilinguis | 77.2 | rock | 20.2 | 20.7 | 35 | 100 | 0,9290 |
| Indonesia | Bukit Baka - Bukit Raya | NA | Staurois | tuberilinguis | 74.1 | branch | 20.2 | 20.7 | 35 | 100 | 0,9290 |
| Indonesia | Bukit Baka - Bukit Raya | NA | Staurois | tuberilinguis | 74.7 | rock | 20.2 | 20.7 | 35 | 100 | 0,9290 |
| Indonesia | Bukit Baka - Bukit Raya | NA | Staurois | tuberilinguis | 70.6 | rock | 20.2 | 20.7 | 35 | 100 | 0,9290 |
| Indonesia | Bukit Baka - Bukit Raya | NA | Staurois | tuberilinguis | 79.4 | rock | 20.2 | 20.7 | 35 | 100 | 0,9290 |
| Malaysia | Kinabalu NP | 0067H | Staurois | tuberilinguis | 45.2 | rock | 19.7 | 17.9 | 45 | 826 | 0,0338 |
| China | Badagong Shan | 2005 | Xenophrys | sangzhiensis | 68.1 | leaf | 22.4 | 21 | 10 | 200 | 0,6667 |
